# Malignant Phyllodes Tumor With Chondroblastic Osteosarcomatous Differentiation: A Case Report

**DOI:** 10.7759/cureus.63915

**Published:** 2024-07-05

**Authors:** Matthew Carpenter, Shahla Masood, Swati Sharma, Raafat Makary, Parlyn Hatch, Noor Marji

**Affiliations:** 1 Pathology, University of Florida College of Medicine – Jacksonville, Jacksonville, USA; 2 Pathology and Laboratory Medicine, University of Florida College of Medicine – Jacksonville, Jacksonville, USA; 3 Radiology, University of Florida College of Medicine – Jacksonville, Jacksonville, USA; 4 Neuropathology, University of Florida College of Medicine – Jacksonville, Jacksonville, USA; 5 Women's Imaging/Radiology, University of Florida College of Medicine – Jacksonville, Jacksonville, USA

**Keywords:** metaplastic breast cancer, breast cancer, metastatic metaplastic breast cancer, breast, brain lesion, brain metastasis, breast pathology, heterologous mesenchymal differentiation, osteochondrosarcomatous differentiation, late metastasis

## Abstract

Malignant phyllodes tumors (MPTs) represent the most pernicious type of intralobular stromal proliferation known as a “fibroepithelial lesion” (FEL). They comprise a small fraction of breast malignancies and can present as either a pure MPT or sometimes include a heterologous component (liposarcoma, chondrosarcoma, osteosarcoma, or rhabdomyosarcoma). Of the fraction of MPTs that include heterologous components, very little about those with chondroblastic osteosarcomatous differentiation has been described in the literature. As such, a characteristic staining profile has yet to be established, even though morphological analysis is the cornerstone of diagnosis. The few reported cases have described a poor prognosis. Therefore, we present a case of MPT with chondroblastic osteosarcomatous differentiation to contribute to the dearth of literature examining this entity.

## Introduction

Phyllodes tumors (PTs) are an uncommon, more cellular variant of biphasic lesions arising in the intralobular stroma of the breast, which are known as “fibroepithelial lesions” (FELs). FELs are thought to exist along a spectrum, which is supported by the fact that they all share mutations in the mediator complex subunit 12 (MED12) gene [1-3], where the least cellular and most benign are fibroadenomas, and the most cellular are malignant phyllodes tumors (MPTs). PTs are rare, accounting for less than 1% of mammary gland tumors [3-4]. Of the three subtypes of PT (benign, borderline, malignant), malignant PTs account for 10% to 30% of PTs [3-4].

MPTs demonstrate the highest degree of stromal cellularity among FELs. They demonstrate “stromal overgrowth,” defined as the absence of epithelial elements in one low-power microscopic field containing only stroma. Nuclear atypia and a mitotic rate of >10 mitoses/10 high-power fields are additionally required to diagnose MPT [3]. Alternatively, the presence of heterologous sarcomatous elements within the tumor, such as chondrosarcoma or osteosarcoma, directly qualifies PTs as malignant regardless of other histopathologic features [4].

With this latter route to the diagnosis of MPT in mind, extraskeletal osteosarcomas are comparably rare, representing less than 1% of soft tissue sarcomas. In mammary sarcomas, primary osteosarcomas are one of the least common sarcomas of the breast [5]. One of the largest reviews of primary osteogenic sarcoma of the breast analyzed a mere 50 cases [6]. As such, literature describing MPTs with chondroblastic osteosarcomatous differentiation is limited to only a few case studies [2]. In this article, we present a case of MPT with a heterologous element of chondroblastic osteosarcomatous differentiation, along with a brief review of the available relevant literature.

## Case presentation

**Figure 1:**
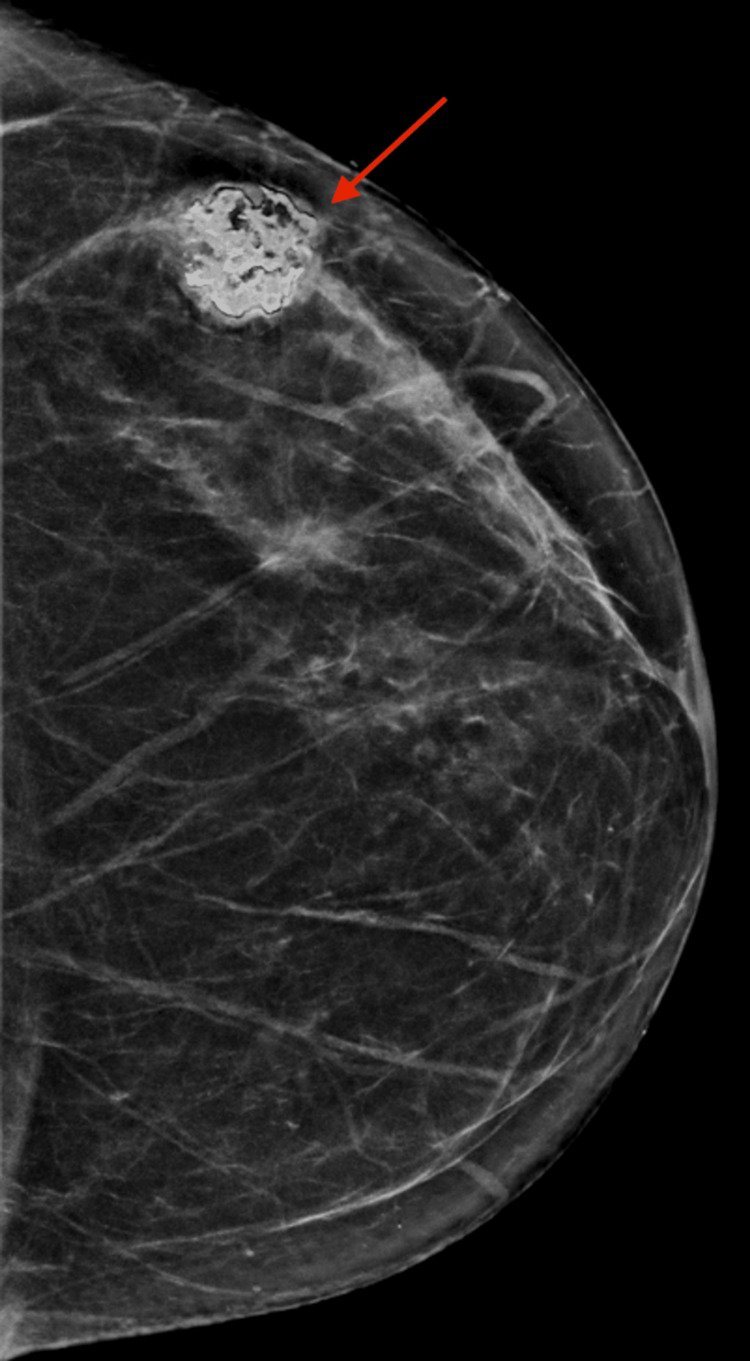
Left breast diagnostic mammogram demonstrates an upper outer calcified mass with lobulated margins (red arrow) and surrounding halo artifact underneath the palpable marker (BI-RADS 4A).

**Figure 2:**
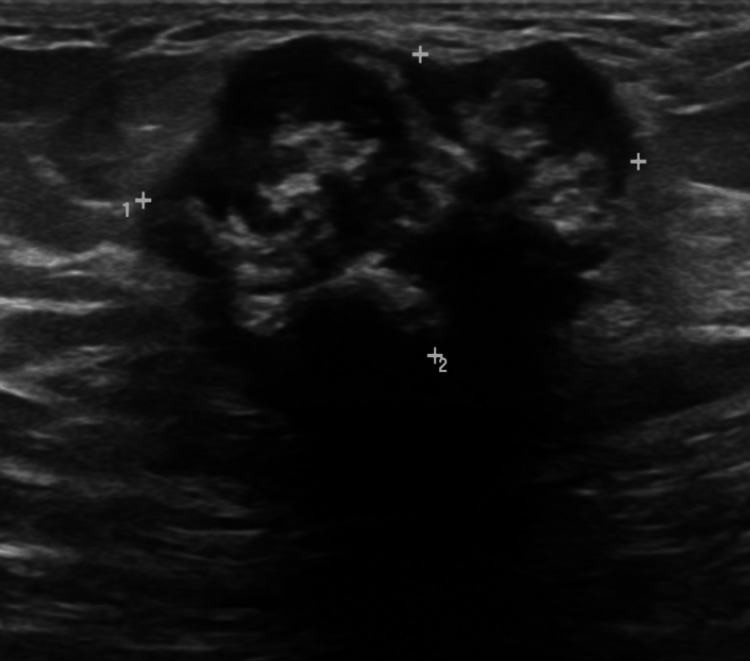
Targeted ultrasound of the left breast demonstrates a hypoechoic mass measuring 3 × 1.8 × 2.7 cm at two o’clock, 6 cm from the nipple, with lobulated margins, dense calcifications, and posterior shadowing (BI-RADS 4A).

A 62-year-old woman with no known family history of breast cancer presented to her primary care provider (PCP) in February 2022 after noticing a lump in her left breast for the previous two weeks. A bilateral diagnostic mammogram and breast ultrasound later that month revealed a partially calcified mass at the two o’clock position, 6 cm from the nipple of the left breast, which measured 1.9 × 1.4 × 1.9 cm corresponds to a densely calcified mass seen on mammography (assigned BI-RADS 4A). In July 2022, that same mass showed an increase in size, which now measures 3.0 × 1.8 × 2.7 cm (Figure 1 and Figure 2.) An ultrasound-guided core biopsy was performed and showed a spindle cell neoplasm with chondro-osseous metaplasia suggestive of an MPT.

**Figure 3:**
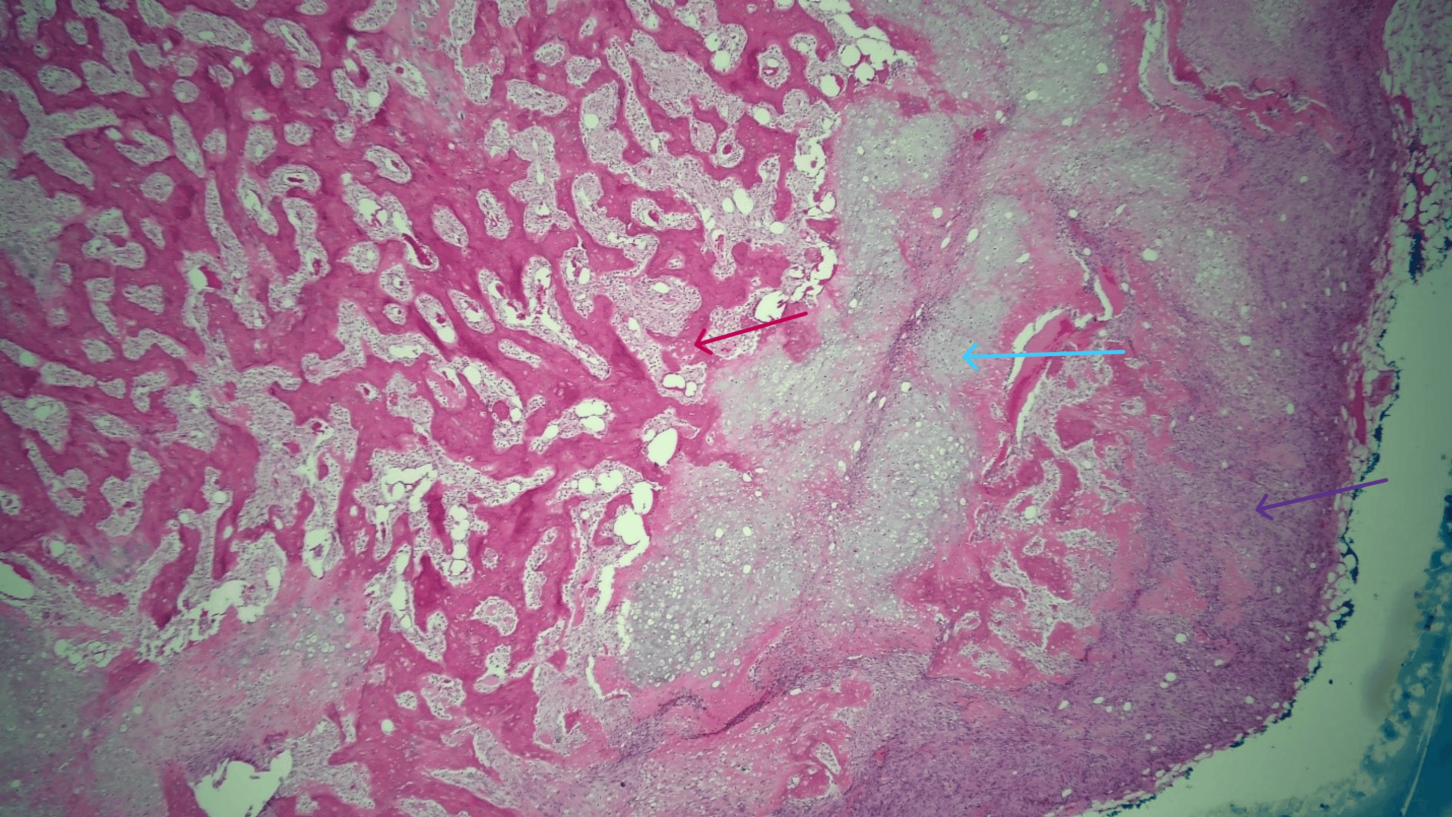
Low-power view of bone (red arrow) and cartilaginous tissue (blue arrow) in a background of spindle cells (purple arrow), comprising a malignant phyllodes tumor with chondroblastic osteosarcomatous differentiation.

**Figure 4:**
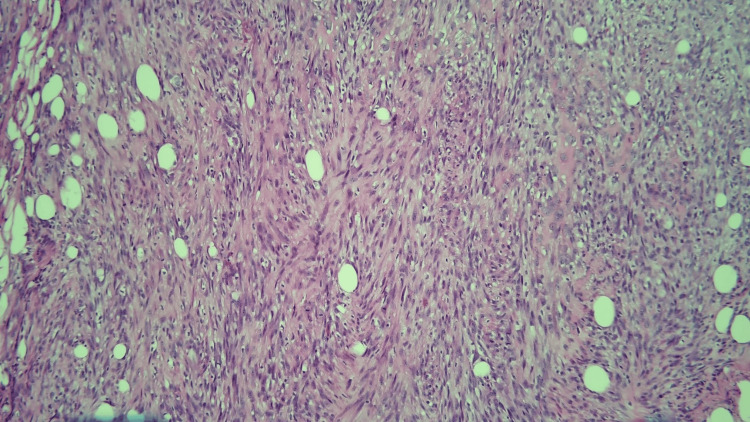
High-power view of the spindle cell component of the tumor.

A subsequent lumpectomy of the left breast mass at the two o’clock position revealed an MPT with chondroblastic differentiation (Figure 3 and Figure 4) that measured 4 cm in the greatest dimension, making it a pathologic stage pT1 tumor. The tumor was present at the superior margin. However, re-excision showed no residual tumor cells. No role for adjuvant therapy (including chemotherapy) was deemed pertinent in this case. The patient is scheduled to have yearly follow-ups with magnetic resonance imaging of the breast. While genetic testing was never performed, there are several genetic alterations that have been observed in tumors such as this one (Table 1).

**Table 1:** Molecular findings in fibroepithelial lesions of the breast BCOR: BCL-6 corepressor gene, EGFR: epidermal growth factor receptor, ERBB4: Erb-B2 receptor tyrosine kinase 4, FA: fibroadenoma, FEL: fibroepithelial lesion, FLNA: filamin A, MAP3KQ: mitogen-activated protein kinase 3, MED 12: mediator complex subunit 12, MLL2/KMT2D: mixed lineage leukemia 2, NF-1: neurofibromatosis type 1, PIK3CA: phosphatidylinositol-4,5-bisphosphate 3-kinase catalytic subunit alpha, PT: phyllodes tumor, RARA: retinoic acid receptor, alpha, RB 1: retinoblastoma 1, SETD2: SET domain containing 2, TERT: telomerase reverse transcriptase

Molecular alteration	Specific manifestations observed
Chromosomal abnormalities	Loss chromosome 13q, gain 1q. Gain of 4q12 important for progression of histologically benign to more atypical higher-grade PTs
MED12	Most prevalent recurrent molecular alteration found in FEL.
TERT	Some studies found TERT promoter mutations primarily occur in PTs and not in FAs. Stromal TERT expression prominently elevated in borderline and malignant PTs vs. benign PTs. TERT promoter mutations most commonly identified within borderline PTs. When synchronous FELs present, TERT upregulated in PT component but not in the clonally related FA presumed precursor lesion.
Other genes	FLNA, SETD2, MLL2, BCOR, MAP3KQ, KMT2D: more mutated in PTs than FAs. NF-1, RB1, PIK3CA, EGFR, TP53, ERBB4: mutated in PTs, though more commonly in borderline and malignant than in benign PTs. RARA: associated with MED12 mutations and influences estrogen signaling pathways; most commonly found in FAs and low-grade PTs. 16-gene NGS panels developed by Sim and colleagues have been designed for the specific characterization of FELs.
Molecular alteration	Specific manifestations observed.

## Discussion

PTs are de-novo lesions originating from periductal and specialized lobular stroma [2]. They are closely related to fibroadenomas, which are collectively considered along a spectrum of FEL, with fibroadenomas at the most benign end and malignant PTs at the opposite end. Bolstering the validity of this spectrum is the presence of MED12 mutations. MED12 mutations have been identified in the stromal component of fibroadenomas. The same mutation is also prevalent in PTs, with 65.1% of benign, 65.6% of borderline, and 42.8% of MPTs possessing it, respectively [7].

Grading of PT involves a group of microscopic parameters (stromal hypercellularity, atypia, mitotic rate, overgrowth, and nature of tumor borders), which are used to divide PT into benign, borderline, and malignant categories. However, the interpretation of some of these parameters, such as the degree of atypia present or the nature of tumor borders, does not have standardized objective criteria devised that can achieve uniformity among pathologists. In addition to this obstacle to incontrovertible grading, PT tends to show intratumoral heterogeneity, where some foci are consistent with a "benign" grade, and others are more consistent with a "borderline" or "malignant" designation [2]. An example of a practical approach sometimes used to categorize PTs is to consider only a tumor displaying all of the histological parameters as distinctly malignant in order to classify the tumor as "malignant" and to consider anything less as "borderline" until there are no features of malignancy, in which case the tumor would be considered "benign" [2].

One of the major criteria used to grade the severity of PTs is the presence of any malignant heterologous elements, which by itself are enough to classify the tumor as a malignant PT, with one notable exception: when the only malignant heterologous component present is a well-differentiated liposarcoma. When well-differentiated liposarcoma occurs as the sole heterologous element in a PT, there is evidence to suggest the metastatic risk is low. Therefore, it is recommended that other malignant stromal features also be present, in addition to the well-differentiated liposarcomatous component, in order to diagnose an MPT [8]. In our case, all microscopic parameters were present, in addition to the rare heterologous elements of chondroblastic osteosarcomatous differentiation. With these findings in hand, the tumor was clearly an MPT.

If the presence of the characteristic leaf-like fibrous stroma and epithelial glands of PTs was not present in our case, however, then it would have been incumbent upon us to rule out another potential disease: primary pure osteosarcoma of the breast. In general, extraskeletal osteosarcomas account for <1% of soft tissue sarcomas and are known to more often localize in soft tissues of the lower extremities [9]. Mammary sarcomas make up less than 1% of all primary breast malignancies, with primary breast osteosarcomas accounting for around 12.5% of these [9, 10]. Tumors with the following criteria are considered “pure osteosarcoma”: absence of bone origin, presence of osteoid or bony matrix, and absence of epithelial differentiation [6]. Since our case did not meet all three of these preconditions, this remarkably rare etiology was not germane to our work-up.

Immunohistochemical studies also play an important role in differentiating malignant PTs from their major differential: primary breast osteosarcomas and metaplastic carcinomas. The presence of diffuse cytokeratin or p63 in the malignant spindle cells is supportive of metaplastic carcinoma [11]. However, caution is warranted in cases of focal keratin or p63 expression, which are also present in PT [12]. CD34 reactivity, which is well described in the stromal cells of PTs, has been reported to be inversely related to adverse histological features [13]. Conversely, CD117 shows increased expression in higher-grade PTs [13].

Any discussion of methods to accurately distinguish the specific type of a FEL would be inadequate without acknowledging how the current armamentarium is woefully unreliable for this task; it would also be incomplete without describing current attempts to gain traction in this endeavor by utilizing putative molecular signatures specific to each subtype of FEL. Attempts to identify molecular markers that can distinguish different subtypes of FELs have been recently summarized by Dadmanesh et al. [14]. While the molecular pathogenesis of FELs in the breast is still not fully understood, there are some promising avenues of research that could allow for more objective classification and clinical guidance of these lesions than the subjective histological landscape currently provides. A summary of current molecular findings for FELs is seen in Table 1.

In our case the spindle cells were negative for AE1/AE3, CK34βE12, SMA, desmin, BLC2, CD34, CK5/6, p63, and β-catenin and CD117. Regardless of the staining pattern, the histological assessment remains the cornerstone in the diagnosis of PT, and adequate sampling is mandatory [2].

## Conclusions

PTs are a rare breast lesion, and their malignant subtypes are uncommon among them. MPTs with heterologous differentiation are rarer still, and those with chondroblastic osteosarcomatous differentiation are exceedingly rare. As such, a distinct staining profile and the effect this phenomenon may have on prognosis and treatment is unestablished. We hereby contribute an important case study of MPT with chondroblastic osteosarcomatous differentiation to the current literature. More work needs to be done to better understand this unique phenomenon.
